# The soybean-derived peptide lunasin inhibits non-small cell lung cancer cell proliferation by suppressing phosphorylation of the retinoblastoma protein

**DOI:** 10.18632/oncotarget.3080

**Published:** 2014-12-31

**Authors:** Elizabeth J. McConnell, Bharat Devapatla, Kavitha Yaddanapudi, Keith R. Davis

**Affiliations:** ^1^ Owensboro Cancer Research Program, Mitchell Memorial Cancer Center, Owensboro, Kentucky; ^2^ James Graham Brown Cancer Center, University of Louisville School of Medicine, Louisville, Kentucky; ^3^ Department of Medicine, University of Louisville School of Medicine, Louisville, Kentucky; ^4^ Department of Pharmacology & Toxicology, University of Louisville School of Medicine, Louisville, Kentucky

**Keywords:** bioactive peptides, lunasin, lung cancer, cell cycle regulation

## Abstract

Lunasin, a soybean bioactive peptide, has both chemopreventive and chemotherapeutic activities. The aim of this study was to determine the chemotherapeutic potential of lunasin against human lung cancer. Treatment of non-small cell lung cancer (NSCLC) cells with highly purified soybean-derived lunasin caused limited, cell-line specific anti-proliferative effects on anchorage-dependent growth whereas two normal bronchial epithelial cell lines were unaffected. Lunasin's antiproliferative effects were potentiated upon utilization of anchorage-independent conditions. Furthermore, NSCLC cell lines that were unaffected by lunasin in anchorage-dependent assays exhibited a dose-dependent inhibition in colony formation or colony size. Mouse xenograft studies revealed that 30 mg lunasin/kg body weight per day decreased NSCLC H1299 tumor volume by 63.0% at day 32. Mechanistic studies using cultured NSCLC H661 cells showed that lunasin inhibited cell cycle progression at the G1/S phase interface without inducing apoptosis. Immunoblot analyses of key cell-cycle proteins demonstrated that lunasin altered the expression of the G1 specific cyclin-dependent kinase complex components, increased levels of p27Kip1, reduced levels of phosphorylated Akt, and ultimately inhibited the sequential phosphorylation of the retinoblastoma protein (RB). These results establish for the first time that lunasin can inhibit NSCLC proliferation by suppressing cell-cycle dependent phosphorylation of RB.

## INTRODUCTION

Lung cancer remains the number one cause of cancer-related deaths among both men and women in the United States, with an estimated 159,260 deaths and 224,210 new diagnoses being expected in 2014. Of those diagnosed, approximately 60% will die within a year. Non-small cell lung cancer (NSCLC) accounts for approximately 84% of all incidences, while small cell lung cancer accounts for 14% [1]. While greater than 90% of the incidence of small cell lung cancer is the result of unchecked proliferation due to acquired mutations in *RB1*, development of NSCLC is attributed to the accumulation of mutations in genes involved in the upstream regulation of the retinoblastoma protein (RB), such as *TP53* (encoding the tumor protein p53), *CCND1* (encoding the G1/S-specific cyclin D1), and *CDKN2A* (encoding the cyclin-dependent kinase inhibitor (CDKI) p16^INK4a^) [2].

Soybean has long been recognized as an important source of high quality food protein. Soy-derived products have received increasing interest due to their purported health benefits, including cardiovascular health, weight management, diabetes, osteoporosis, and cancer prevention. [3-9] Additionally, epidemiological observations have identified a correlation between high levels of soybean consumption with lowered incidence and mortality due to breast, prostate, colon and lung cancer [5, 10-17].

Lunasin, a 43-44 amino acid peptide derived from soybean, consists of nine consecutive aspartic acid residues at the C-terminus, a RGD cell adhesion motif and a helical region exhibiting structural homology to conserved sequences of chromatin binding proteins [18-20]. Although lunasin has been identified in a number of other plants, including barley, wheat, black nightshade (*Solanumnigrum L*.), rye and the ancient Aztec grain amaranth [21-24], recent studies have suggested that lunasin is not naturally found in cereals [25, 26]. Lunasin has the ability to suppress transformation in mammalian cells by oncogenes (E1A- and *ras*-mediated) and chemical carcinogens 7,12-dimethylbenz(a)anthracene (DMBA) and 3-methylcholanthrene, as well as DMBA-induced skin tumorigenesis in a mouse model [20, 27-30]. Further studies have suggested that lunasin exhibits antiproliferative effects on some established cancer cell lines, including leukemia, breast and colon [5, 30-32], as well as acting as an anti-inflammatory in lipopolysaccharide-activated macrophages [27, 33]. Lunasin has been shown to be effective at reducing tumor growth in mouse xenograft models of breast and colon cancer [30, 31]. More recently, lunasin has been shown to be able to enhance cytokine-induced anti-tumor immunity via activation of natural killer and dendritic cells [34]. These data strongly suggest that lunasin exhibits both chemopreventive and chemotherapeutic properties and is active against a broad array of cancer types.

One limitation of the *in vitro* studies with lunasin over the past decade is that they have been performed under anchorage-dependent growth conditions. Although providing a convenient and economical means for the study of mammalian cells, plastic substrates commonly used for anchorage-dependent cell culture are not representative of cellular environments found within organisms, resulting in the loss of cell-specific architecture as well as mechanical and chemical cell-cell communication. In addition, the majority of the studies were performed using different forms of lunasin including a synthetic lunasin peptide, lunasin enriched soy flour, lunasin-like peptides or a mixture of peptides, rather than a highly purified lunasin isolated from a natural source. The aim of this study was to evaluate lunasin's effect on the proliferation of NSCLC both *in vitro* and *in vivo* utilizing highly purified lunasin (>99% purity) isolated from soybean white flake [18]. Our results show for the first time that the *in vitro* effects of lunasin on NSCLC is significantly higher in anchorage-independent assays, and correlates significantly with its *in vivo* effects in a NSCLC mouse xenograft model. Mechanistic studies demonstrate that the inhibition of NSCLC proliferation by lunasin is the result of a combination of alterations in the expression of the cyclin-dependent kinase (CDK) complex components cyclin D1, CDK4 and CDK6 and the timing of Akt activation by phosphorylation at S473, which acts as a negative regulator of p27^Kip1^ expression. Ultimately, this results in suppression of RB phosphorylation and inhibition of cell cycle progression.

## RESULTS

### Lunasin exhibits cell-line specific anti-proliferative activity

**Figure 1:**
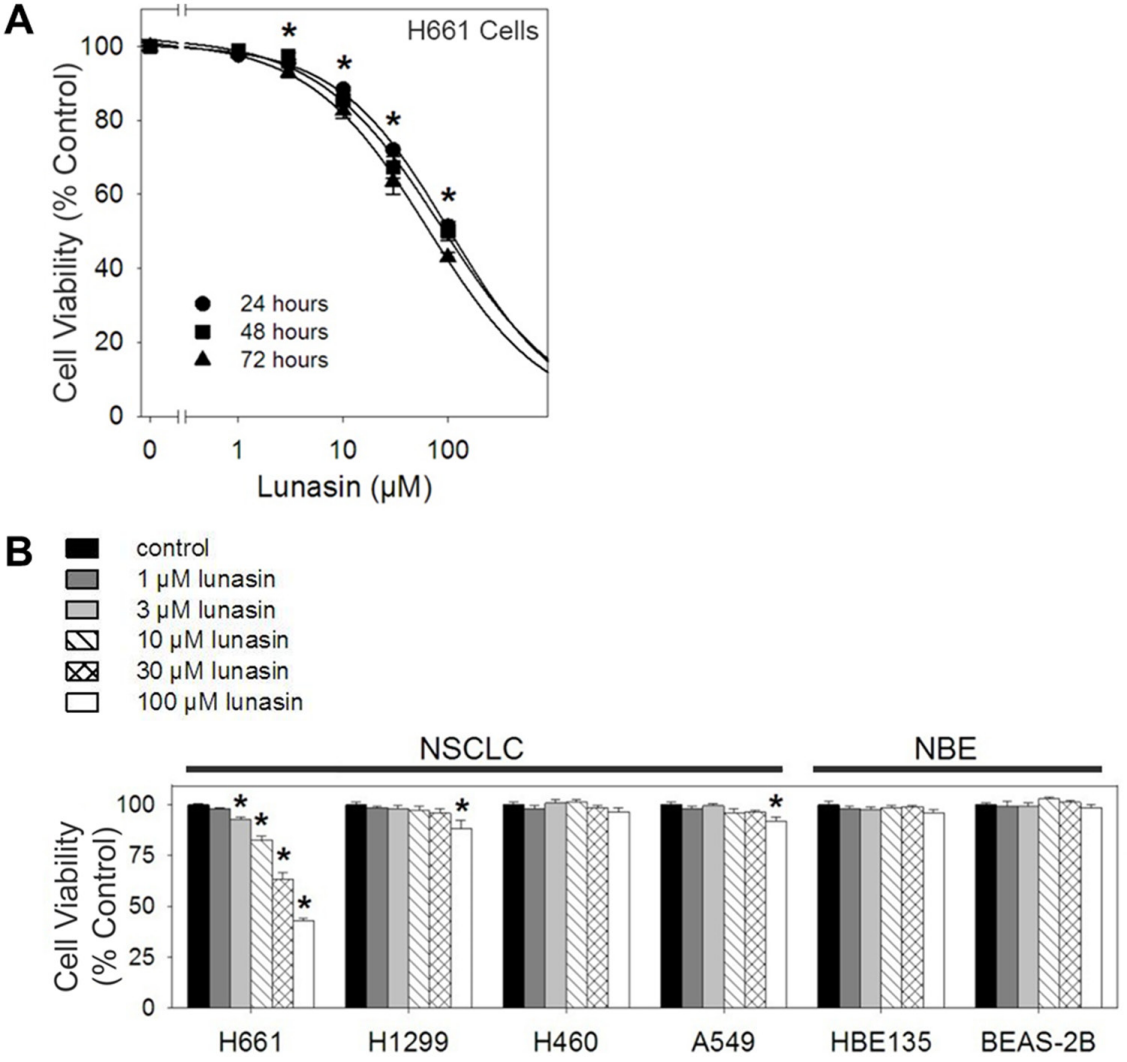
Lunasin exhibits cell-line specific anti-proliferative activity on NSCLC cells under anchorage-dependent conditions MTS cell proliferation assays were performed under anchorage-dependent conditions in the presence of vehicle or various concentrations of lunasin (1 – 100 μM) over 24, 48 and 72 hours, re-dosing every 24 hours. A) H661 exhibited a dose-dependent inhibition of proliferation over 24, 48 and 72 hours. B) Treatment of other established NSCLC cell lines and the NBE cell lines HBE135-E6E7 and BEAS-2B resulted in little to no effect when treated with lunasin over 3 days, 72 hour data shown. Data represented as ± SD of the mean for at least three independent experiments conducted in quintuplicate. The asterisk (*) indicates a significant (*p* < 0.001) decrease in proliferation when compared to the control. For Fig. 1A, the significant difference applies to the dose-response of all three incubation times.

Exposure to lunasin over 24 to 72 hours resulted in a dose-dependent inhibition of proliferation in H661 NSCLC cells when grown under anchorage-dependent conditions (Fig. 1A). At 100 μM lunasin, proliferation was inhibited by 48.9%, 51.1% and 57.7% after 24, 48 and 72 hours respectively, with estimated 50% inhibitory concentrations (IC_50_) of 103.1 μM, 86.8 μM and 63.9 μM, respectively. However, lunasin treatment of other NSCLC cell lines (H1299, H460 and A549) and NBE cell lines (HBE135-E6E7 and BEAS-2B) resulted in little or no effect when treated over 72 hours (Fig. 1B). These results indicate that lunasin exhibits cell-line specific anti-proliferative activity on human NSCLC cells grown under anchorage-dependent conditions and that lunasin does not have any obvious detrimental effects on NBE cells.

### Lunasin inhibits anchorage-independent growth in multiple cell lines

**Figure 2:**
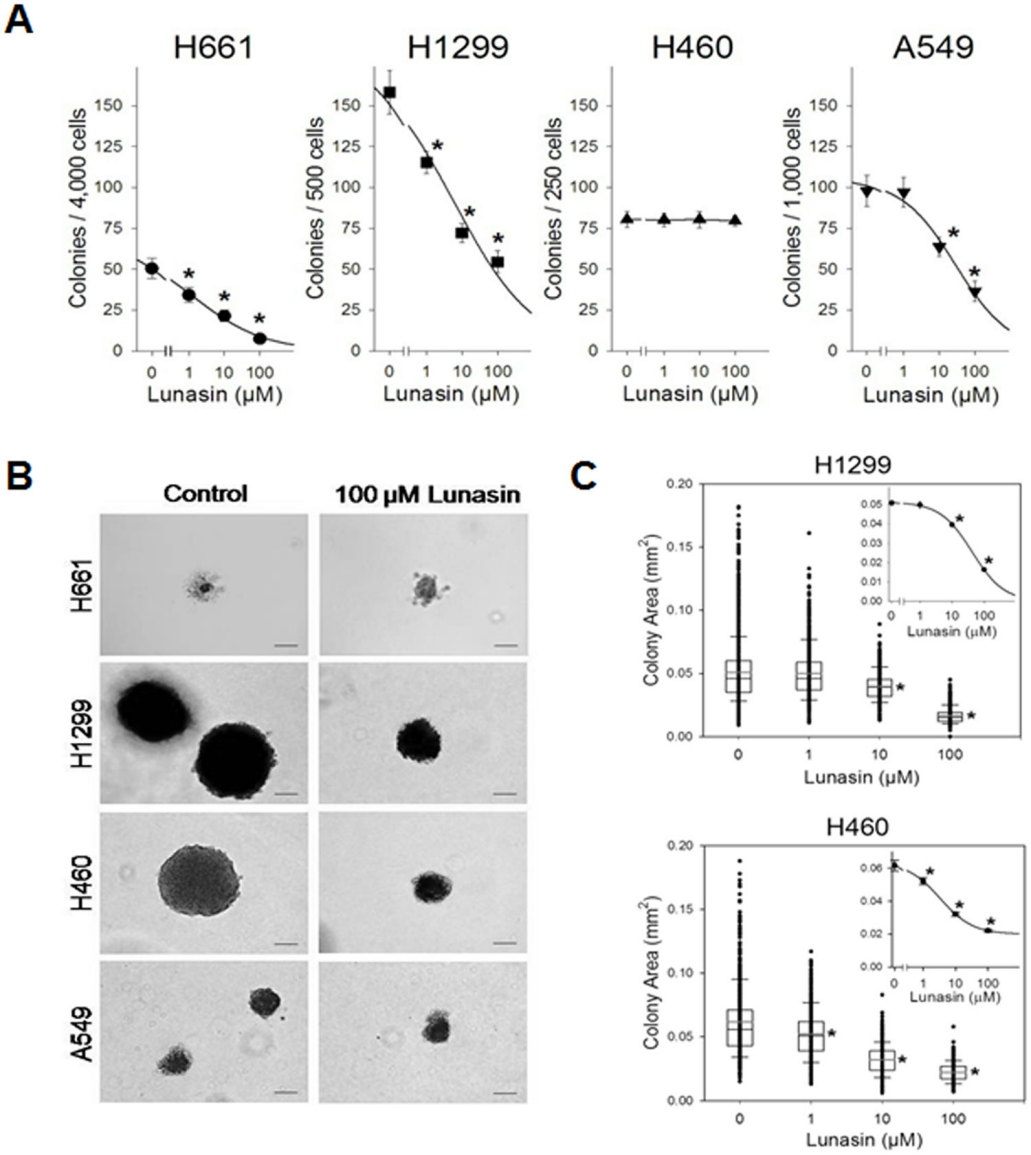
Lunasin inhibits anchorage-independent growth of NSCLC cells Drug sensitivity anchorage-independent growth assays were performed in presence of vehicle or various concentrations of lunasin (1 – 100 μM) on established NSCLC cell lines in order to assess its effects upon proliferation and growth. A) H661, H1299 and A549 cells all exhibited a dose-dependent inhibition in colony formation, while H460 cells exhibited no change in colony number. B) Representative 40X images of crystal violet stained anchorage-independently grown colonies. Scale bar represents 0.1 mm. C) H1299 and H460 cells exhibited a dose-dependent reduction in colony size under anchorage-independent growth conditions. Colonies (> 100 μm) were evaluated using ImageJ software v1.45. Data represented as ± SD of the mean for at least three independent experiments in quadruplicate. The asterisk (*) indicates a significant (*p* < 0.001) decrease in colony number or colony size (mm^2^) when compared to the control.

Anchorage-dependent growth conditions are self-limiting in that they result in the loss of the normal cell environment and do not mimic the tumor environment *in vivo*. Hence we surmised that the lunasin's ability to inhibit anchorage-independent growth would represent a more meaningful indication of its potential as an anti-proliferative agent on human NSCLCs. Of the four human NSCLC cell lines tested, all but one, H460, exhibited a significant decrease in the number of colonies formed in soft agar (Fig. 2A). H661, H1299 and A549 cells all exhibited a dose-dependent decrease in colony formation upon exposure to lunasin, with 100 μM lunasin resulting in 85.3% (IC_50_1.3 μM), 65.6% (IC_50_6.0 μM) and 62.7% (IC_50_28.8 μM) inhibition of colony formation respectively. For H661 cells, the IC_50_ decreased 49-fold from 63.9 μM lunasin (anchorage-dependent) to 1.3 μM lunasin (anchorage-independent), clearly demonstrating an increase in sensitivity to lunasin under anchorage-independent conditions.

Although H460 cells showed no decrease in the total number of colonies formed upon lunasin treatment, they did exhibit a dose-dependent decrease in colony size when exposed to lunasin in soft agar, as did H1299 cells (Fig. 2B, Fig. 2C). H460 cells exhibited a 64.0% decrease in colony size upon treatment with 100 μM lunasin, while H1299 cells exhibited a 67.7% decrease in colony size. The statistical significance was high (*p* < 0.001) when compared to controls, with 1 μM lunasin treatment being highly significant for H460 colony size reduction and 10 μM lunasin treatment being highly significant for H1299 colony size reduction (Fig. 2C). Both H661 and A549 formed small colonies in soft agar, and their colony sizes were not affected by lunasin exposure (Fig. 2B). These results indicate that lunasin's anti-proliferative activity is enhanced under anchorage-independent conditions, with all NSCL cancer cell lines tested exhibiting an effect on colony number and/or size.

### Lunasin reduces the growth of xenografted NSCLC tumors *in vivo*

**Figure 3:**
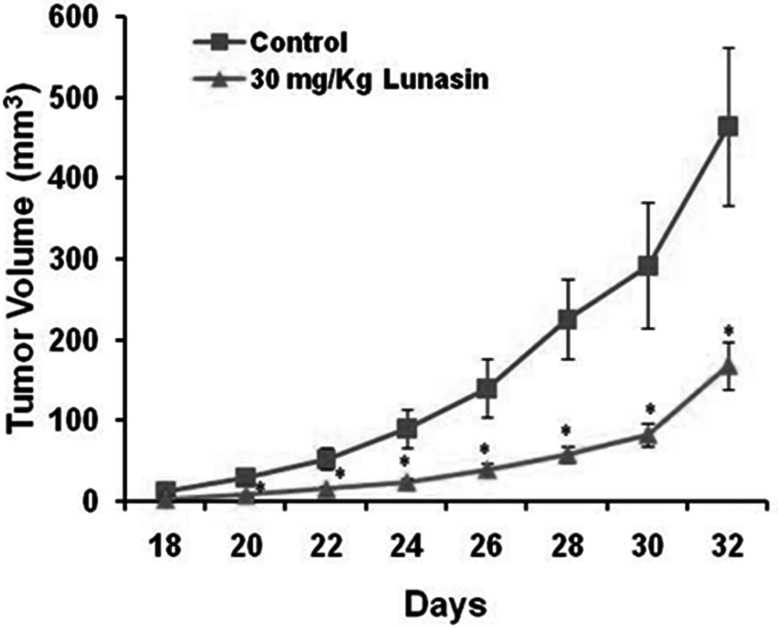
Lunasin reduces NSCLC tumor growth *in vivo* Mouse xenografts using H1299 cells were performed in order to evaluate lunasin's anti-tumorigenic properties *in vivo*. Nude mice were injected with 2 × 10^6^ human H1299 cells, and subsequently subjected to daily IP injections of lunasin 30 mg/kg body weight. Tumor volumes were measured by vernier caliper beginning on day 18 after the initiation of tumors. Data represented as ± SD of the mean. The asterisk (*) indicates a significant (*p* < 0.05) decrease in tumor volume (mm^3^) when compared with vehicle controls.

To evaluate whether the inhibition of *in vitro* anchorage-independent growth of NSCLCs can be recapitulated *in vivo*, we established tumor xenografts by subcutaneous implantation of H1299 cells in nude mice. Cancer cell implantation was followed by concurrent and subsequent daily IP injections of lunasin (30 mg/kg body weight). Whereas all mice in the vehicle control and lunasin treated cohorts exhibited tumor growth, the growth rate and size of tumors differed significantly between the two groups. Tumor growth was palpable in both study groups by day 18 post initiation, but the tumors in the control group grew at a much faster rate. By day 32 post initiation, tumor volume in the control group averaged 455 mm^3^, while tumor volume in the lunasin-treated group averaged 166 mm^3^, representing a decrease of 63% in tumor volume (Fig. 3). This demonstrates a significant inhibition of NSCLC tumors in mice treated with lunasin and represents the first report of lunasin having antitumor effects against NSCLC *in vivo*.

### Lunasin's anti-proliferative activity is not apoptosis-induced

**Figure 4:**
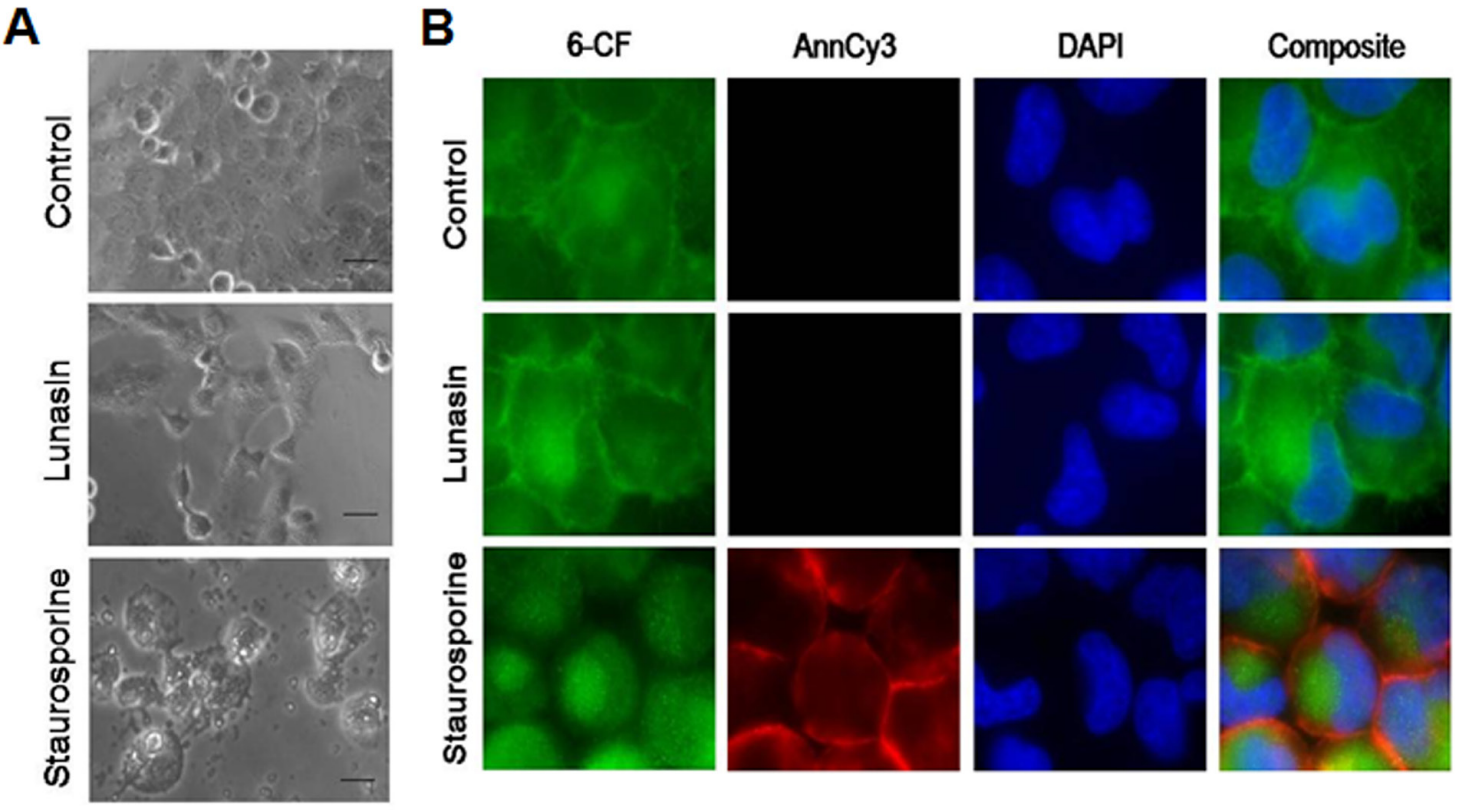
Lunasin's anti-proliferative activity on NSCLC cells is not apoptosis-induced Apoptosis determination in H661 cells was ascertained microscopically by the detection of externalized phosphatidylserine bound by a fluorescent annexin conjugate under anchorage-dependent growth conditions. Cells were grown in the presence of vehicle or 100 μM lunasin for 24 hours or 1 μM staurosporine for 4 to 6 hours (positive control). A) Representative 10X bright-field images showing the absence of characteristic apoptotic morphology in cells treated for 24 hours with lunasin in contrast to the typical apoptotic morphological features, rounding and blebbing, exhibited by cell exposed to staurosporine for 6 hours. Scale bar represents 50 μm. B) Representative fluorescent 40X images showing the absence of annexin detection and lack of characteristic apoptotic morphology in cells exposed to lunasin for 24 hours in contrast to positive annexin detection and the onset of apoptotic morphology (rounding) in cells exposed to staurosporine for 4 hours. Images were analyzed with AxioVision software v4.6.3.0.

Our initial studies demonstrated that lunasin inhibited the proliferation of NSCLC *in vitro* and *in vivo*. To begin determining the mechanisms whereby lunasin inhibits NSCLC cell proliferation, we investigated the molecular basis of lunasin sensitivity in H661 cells grown in adherent culture. We first assessed the effects of lunasin on H661 cells with respect to the induction of apoptosis. Although lunasin exposure resulted in a dose-dependent inhibition of proliferation in H661 cells (Fig. 1A, Fig. 2A), no gross morphological changes were noted that would otherwise suggest apoptosis or necrosis (Fig. 4A). While exposure of H661 cells to 1 μM staurosporine for 6 hours under anchorage-dependent conditions resulted in the characteristic morphological changes associated with apoptosis (rounding and blebbing), treatment of these cells with 100 μM lunasin for 24 hours resulted only in a decrease in cell number (Fig. 4A, Fig. 1A).

To further establish that lunasin's effect on H661 cells is not related to apoptosis, immunofluorescent detection of translocated phosphatidylserine (an early marker of apoptosis) was performed. A double labeling method where an annexin conjugate AnnCy3, binding externalized phosphatidylserine on the plasma membrane, and 6-CFDA, a non-fluorescent compound that enters cells and is subsequently hydrolyzed by esterases in living cells to produce the fluorescent compound 6-carboxyfluorescein (6-CF), was used to discriminate between early apoptotic, necrotic, and living cells. Under this scheme, live cells are only 6-CF positive, necrotic cells are only AnnCy3 positive, and early apoptotic cells are both 6-CF and AnnCy3 positive. As shown in Fig. 4B, H661 cells exposed to 1 μM staurosporine for 4 hours were both 6-CF and AnnCy3 positive, indicating that they are in the throes of early apoptosis. Furthermore, the early apoptotic morphological characteristic of rounding was present in staurosporine treated cells (Fig. 4B composite). However, control cells and cells exposed to 100 μM lunasin for 24 hours were only 6-CF positive, indicating apoptosis was not induced. Additionally, no morphological changes associated with apoptosis were observed (Fig. 4B composite). These results indicate that lunasin's anti-proliferative activity is not significantly mediated by an apoptotic mechanism in H661 cells.

### Lunasin inhibits cell cycle progression at the G1/S phase interface

**Figure 5:**
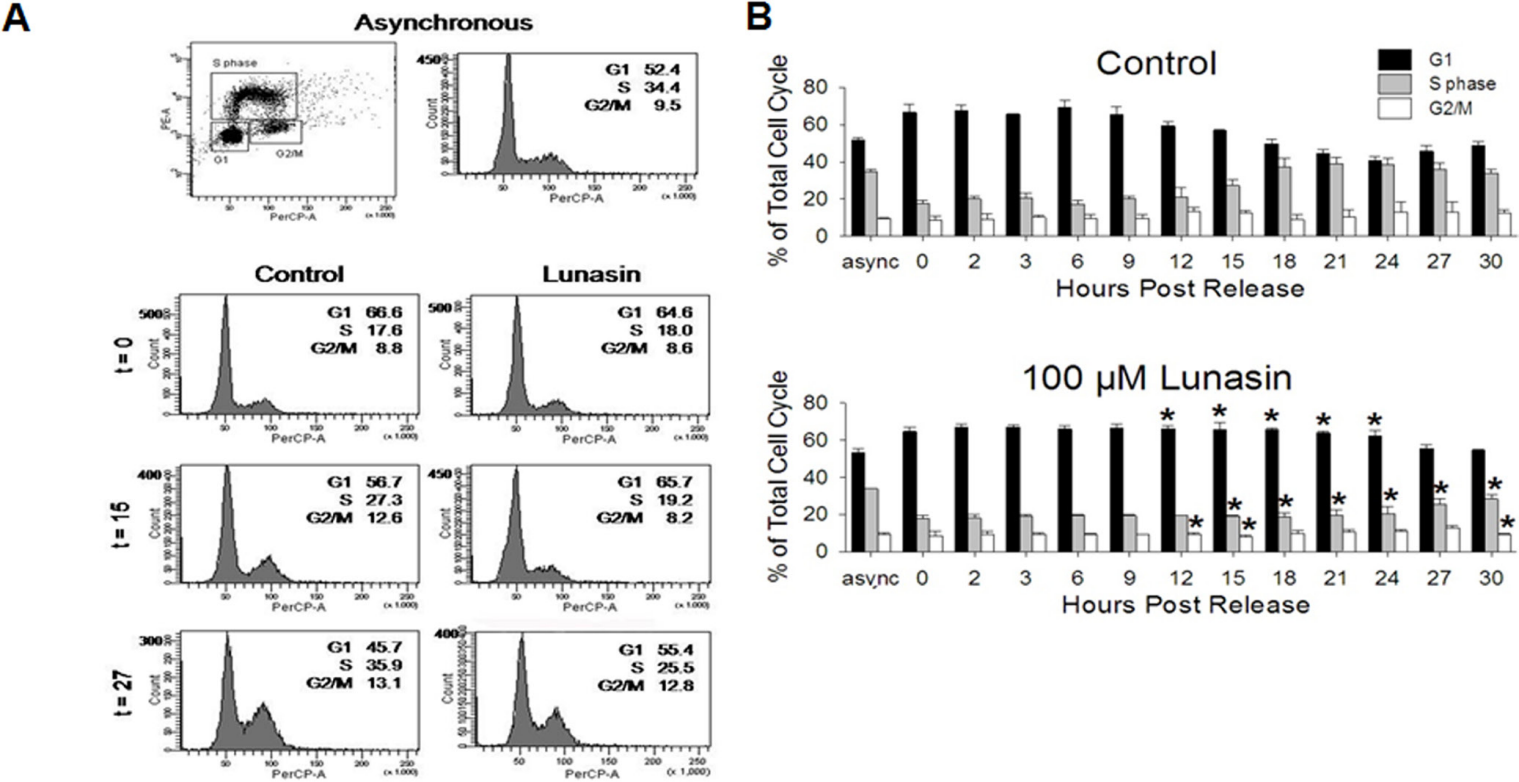
Lunasin inhibits cell cycle progression at the G1/S phase interface BrdU-based cell cycle analyses were performed on H661 cells under anchorage-dependent growth conditions in order to determine if lunasin's anti-proliferative activity is due to alterations in the cell cycle. Cultures were synchronized by serum starvation for 72 hours, treated with vehicle or 100 μM lunasin for 4 hours, and subsequently re-stimulated with medium containing 10 % serum in the presence of vehicle or 100 μM lunasin. After indicated times, cells were pulsed for 30 minutes with BrdU and prepared for analysis by flow cytometry. Asynchronous, pulsed control cells were used for standardization. A) Representative flow cytometry dot blot and histogram for asynchronous cultures and histograms for synchronized vehicle control and lunasin exposed cultures at 0, 15, and 27 hours. B) Corresponding bar graphs illustrating the delay in progression from G1 to S phase due to lunasin exposure. Samples were evaluated using BD FACSDiva Software v6.1.2. Data represented as ± SD of the mean for three independent experiments. The asterisk (*) indicates a significant (*p* < 0.05) difference in the proportion of cells residing in G1,S phase or G2/M of lunasin treated cells when compared to controls at the corresponding time points.

To determine if alterations in the progression of the cell cycle were responsible for the observed anti-proliferative effects of lunasin on H661 cells under anchorage-dependent growth conditions, cell cycle analyses using BrdU incorporation and flow cytometry were performed. Asynchronous cultures of H661 cells exhibited typical cell cycle distribution with the majority of cells residing in G1 at any given time (Fig. 5A). It should be noted that cancer cells, unlike normal cells, have developed mechanisms to avoid quiescence due to the lack of mitogenic stimuli and evade anti-growth signals that would otherwise result in suppression of their growth. Serum starvation for 72 hours resulted in increased numbers of cells residing G1 (52.4% to 66.6%) and a decrease in the cells in S phase (34.4% to 17.6%, Fig. 5A-B). Thus, H661 cells did not exhibit complete synchronization. Synchronized control cultures of H661 cells exhibited an inception to progress from G1 to S phase of the cell cycle at approximately 15 hours post release, as evidenced by the concomitant decrease in the proportion of cells in G1, from 66.6% to 56.7%, and increase in the proportion of cells in S phase, from 17.6% to 27.3% (Fig. 5A-B). Cells treated with 100 μM lunasin exhibited a delay in this inception to progress through the cell cycle (Fig. 5A-B). At 27 hours post release, lunasin exposed cells exhibited the simultaneous decrease in proportion of cells in G1 (64.6% to 55.4%) and increase in proportion of cells in S phase (18.0% to 25.5%). This represents an approximate 12 hour delay in the progression of the cell cycle due to lunasin exposure. These results indicate that the anti-proliferative activity of lunasin on H661 cells is primarily due to an inhibition of the progression of the cell cycle at the G1/S phase interface.

### Lunasin alters the expression and phosphorylation of key proteins involved in the G1/S phase transition

**Figure 6:**
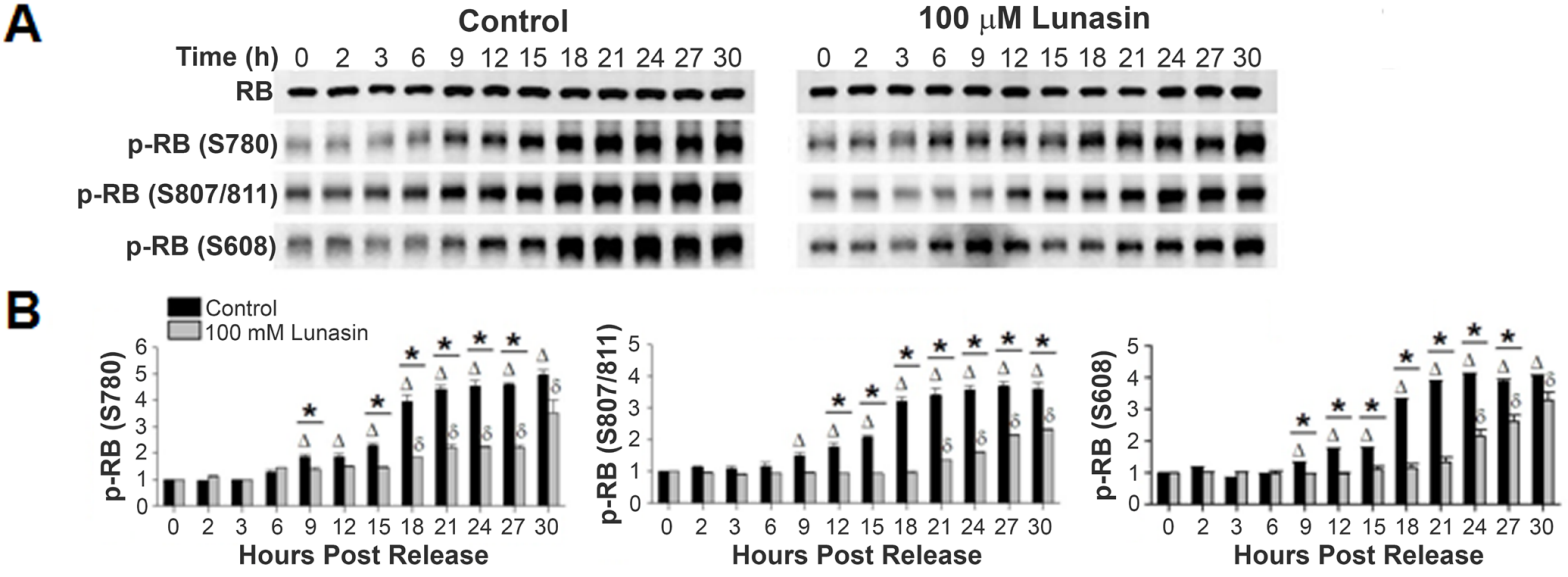
Lunasin alters the phosphorylation of RB Synchronized H661 cultures grown under anchorage-dependent growth conditions were treated with vehicle or 100 μM lunasin for 4 hours, and subsequently re-stimulated with medium containing 10 % serum in the presence of vehicle or 100 μM lunasin. Cells were harvested at the indicated times and protein extracts were isolated and subjected to SDS-PAGE and immunoblot analysis. A) Representative immunoblots of vehicle control and 100 μM lunasin exposed H661 cells probed with antibodies specific for total RB, and RB phosphorylated at S780, S807/811 and S608. β-actin was used for a loading control. B) Corresponding bar graphs of phosphorylated RB showing the delay in onset of RB phosphorylation. Immunoblots were analyzed using ImageJ software v1.45. Data represent the mean ± SD for three independent experiments. The asterisk (*) indicates a significant (*p* < 0.05) difference in protein phosphorylation levels when comparing control and lunasin treated cells at individual time points, while △ (control) and δ (lunasin) indicate a significant (*p* < 0.05) difference compared with the corresponding t = 0 measurement.

To elucidate the mechanism by which lunasin delays progression of the cell cycle at the G1/S phase interface, immunoblot analyses of key cell cycle proteins involved in the transition from G1 to S phase were performed. Since phosphorylation of RB is the key step in initiating progression from G1 to S phase, evaluation of its phosphorylation served as a starting point. Evaluation of various early RB phosphorylation sites revealed a correlation between RB phosphorylation and the progression to S phase for both control and lunasin exposed cells (Fig. 6A). However, an alteration in the timing of this phosphorylation was noted. Whereas RB in control cells began to undergo specific serine phosphorylation by 6 to 9 hours post release, RB phosphorylation in lunasin exposed cells failed to begin until 18, 21, and 24 hours post release at S780, S807/811, and S608, respectively (Fig. 6A-B). This represents a delay in phosphorylation by as much as 15 hours when compared to control cells, indicating lunasin treatment results in a suppression of the phosphorylation of RB.

**Figure 7:**
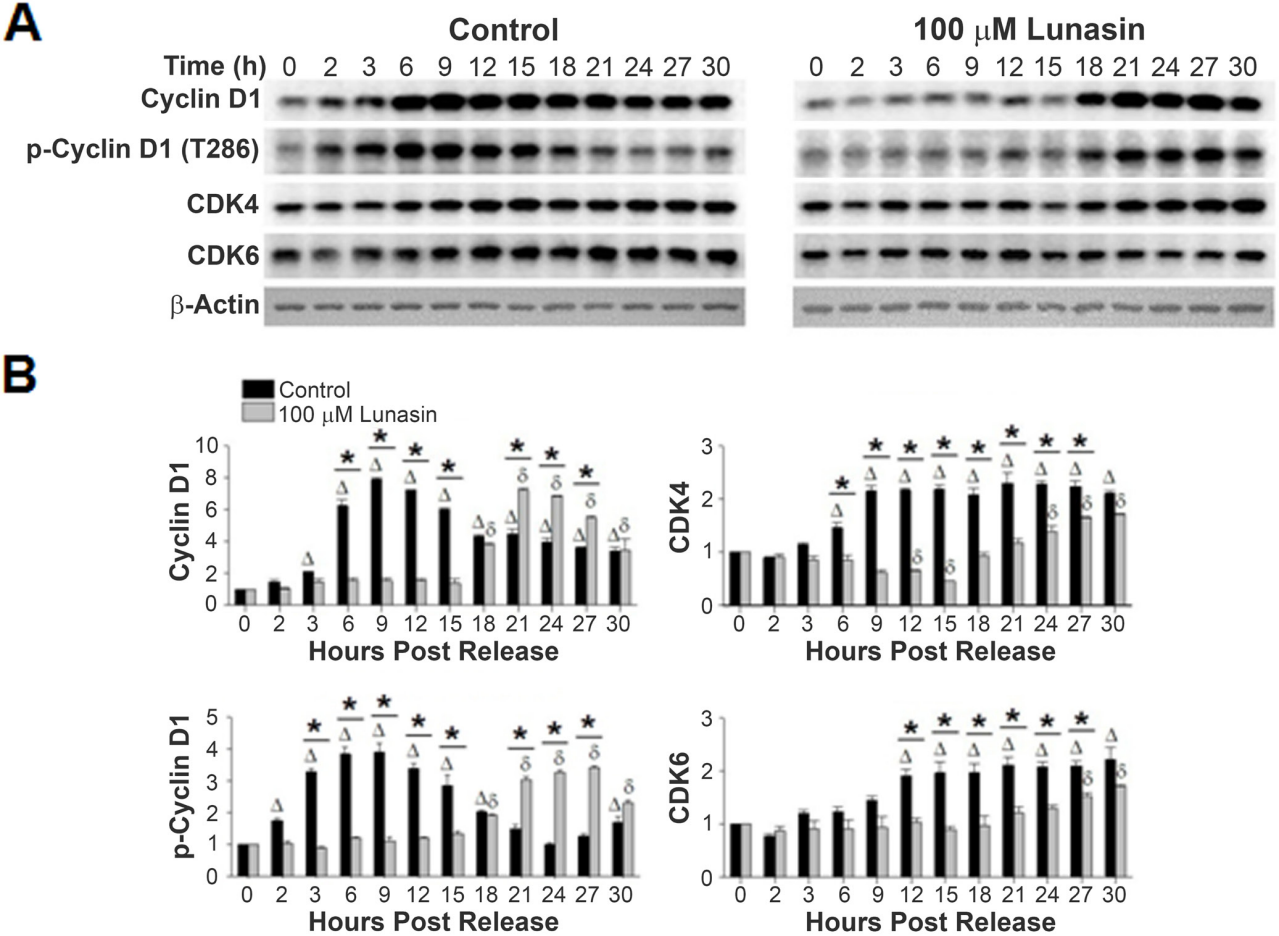
Lunasin alters expression levels of cyclin D1, CDK4, and CDK6 A) Protein extracts from the same experiments described in Fig. 6 were subjected to SDS-PAGE and immunoblot analysis using antibodies specific for cyclin D1, cyclin D1 phosphorylated at T286, CDK4, and CDK6. β-actin was used for a loading control. B) Corresponding bar graphs showing the alterations in expression of the individual subunits of the cyclin D1-CDK 4/6 complex which is responsible for the early phosphorylation of RB and phospho-cyclin D1 (T286). Immunoblots were analyzed using ImageJ software v1.45. Data represent the mean ± SD for three independent experiments. The asterisk (*) indicates a significant (*p* < 0.05) difference in protein phosphorylation levels when comparing vehicle control and lunasin-treated cells at individual time points, while △ (control) and δ (lunasin) indicate a significant (P < 0.05) difference compared with the corresponding t = 0 measurement.

Various cyclin-CDK complexes are responsible for phosphorylating RB throughout the cell cycle, with cyclin D1-CDK 4/6 complex performing the initial phosphorylation in G1. Synchronized control cultures of H661 cells exhibited an increase in the expression of cyclin D1 beginning at 3 hours post release (Fig. 7A-B). By 6 hours, the level of expression had increased 6.3 fold, with a maximum of a 7.9 fold increase noted at 9 hours post release. However, cells released in the presence of lunasin did not exhibit an increase in cyclin D1 until 18 post release, with a maximum 6.4 fold increase noted at 21 hours post release. This represents a delay of 15 hours in the expression of cyclin D1. Analyses of cyclin D1 T286 phosphorylation, which targets cyclin D1 for degradation, demonstrated that although delayed in lunasin-treated H661 cells, the relative timing and level of T286 phosphorylation with respect to cyclin D1 accumulation were similar in control and lunasin-treated cells (Fig. 7A-B). This indicates that the primary effects of lunasin on cyclin D1 accumulation are related to modulating expression and that degradation pathways are not significantly affected.

We observed a similar delay in CDK4 expression in lunasin-treated cells compared to control cells. CDK4 and CDK6 expression in control cells exhibited an increase in expression beginning between 3 to 6 hour post release (Fig. 7A-B). The expression levels increased approximately 2 fold by 9 and 12 hours for CDK4 and CDK6 respectively, and remained relatively stable over the remaining 30 hours post release. However, levels of CDK4 in cells released in the presence of lunasin remained relatively unchanged until approximately 21 hours post release. By 30 hours post release, both kinases exhibited a 1.7 fold increase in their expression levels (Fig. 7A-B). These results indicate that lunasin's suppressive effects on the phosphorylation of RB are due, at least in part, to alterations in the expression of the subunits of the cyclin D1-CDK 4/6 complex.

In addition to transcriptional regulation, cyclin D1/CDK activities are regulated by two families of CDKIs: INK4 and Cip/Kip inhibitors. Synchronized control cultures of H661 cells did not exhibit a change from base level expression of p15^INK4b^, p16^INK4a^, p18^INK4c^ or p19^INK4d^ over 30 hours post release (data not shown). This was also the true for cells released in the presence of lunasin, with no significant difference being noted when compared to controls. Of note, alterations in p16^INK4a^ expression were not expected as H661 cells contain a mutated form of this protein with a reduced half-life [35]. These results indicate that aberrations in the expression levels of the INK4 family of CDKIs are not involved in lunasin's effect on the progression of the cell cycle.

**Figure 8:**
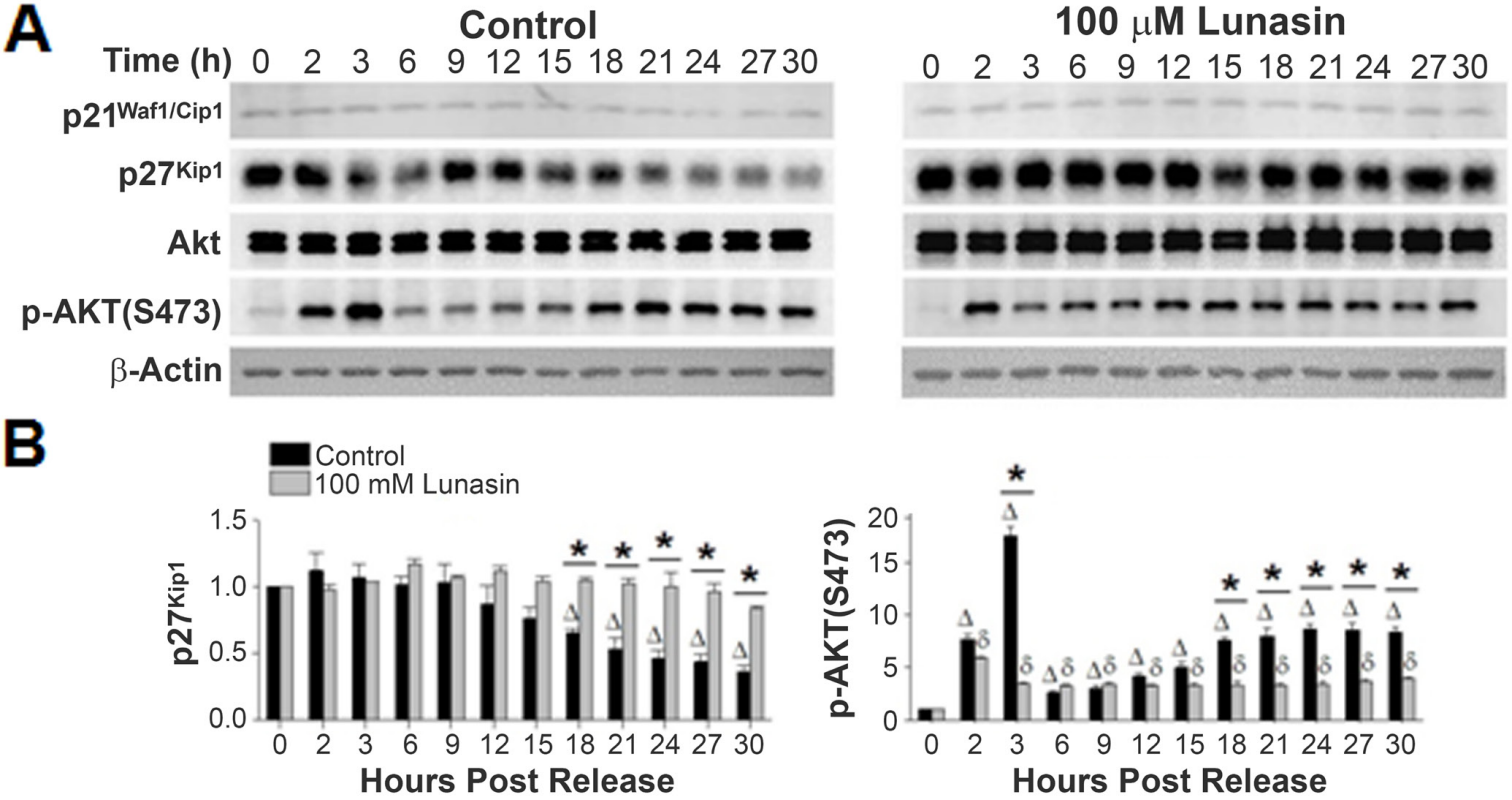
Lunasin alters expression levels of p27Kip1 and phosphorylation of Akt at S473 A) Protein extracts from the same experiments described in Fig. 6 were subjected to SDS-PAGE and immunoblot analysis using antibodies specific for p21^Waf1/Cip1^, p27Kip1, total Akt, and phosphor-Akt (S473). B) Corresponding bar graphs showing alterations in the CDKI p27^Kip1^ expression and in the activation phosphorylation of Akt at S473. Immunoblots were analyzed using ImageJ software v1.45. Data represent the mean ± SD for three independent experiments. The asterisk (*) indicates a significant (*p* < 0.05) difference in protein phosphorylation levels when comparing vehicle control and lunasin-treated cells at individual time points, while △ (control) and δ (lunasin) indicate a significant (*p* < 0.05) difference compared with the corresponding t = 0 measurement.

P21^Waf1/Cip1^ and p27^Kip1^ are also potent CDKIs. Specifically, p21^Waf1/Cip1^ binds to cyclin D1-CDK4 complex (at high stoichiometric ratios), and p27^Kip1^ binds to cyclin D1 or cyclin D1-CDK4 complex, inhibiting their activity. Thus, high expression levels of either CDKI lead to cell cycle arrest in G1. No variations of p21^Waf1/Cip1^ expression levels were observed in synchronized cultures of H661 cells released in the presence of vehicle or lunasin, (Fig. 8A-B). This is not unexpected considering H661 cells express a mutated form of p53, and hence express low, non-inducible levels of p21^Waf1/Cip1^ [36, 37]. On the other hand, expression levels of p27^Kip1^ were significantly altered in lunasin exposed cells when compared to untreated controls (Fig. 8A-B). Synchronized control cultures of H661 cells exhibited an initial decrease in p27^Kip1^ levels by approximately 12 hours post release, with a 2.8 fold lower expression level being noted at 30 hours post release. The declining level of p27^Kip1^ correlates with increased phosphorylation of RB and subsequent progression to S phase in control cells (Fig. 6A). In contrast, p27^Kip1^ levels were elevated in lunasin exposed cells compared to control, and these levels remained constant over 30 hours post release (Fig. 8A-B). These results indicate the suppression of NSCLC cell cycle by lunasin is mediated through the stabilization of the CDKI p27^Kip1^.

To further delineate lunasin's effect upon the levels of p27^Kip1^, the expression and timing of Akt activation by phosphorylation at S473, which subsequently acts as a negative regulator of p27^Kip1^ expression, were evaluated [38-41]. Synchronized cultures of both control and lunasin treated H661 cells exhibited very similar, steady state levels of Akt (Fig. 8A-B). However, when phosphorylation of Akt at S473 was examined, control cultures exhibited an initial jump at 2 hours post release, representing a 7.7 fold increase in phosphorylation (Fig. 8A-B). This jumped to a 17.5 fold increase at 3 hours post release before re-stabilizing at 6 hours post release to approximately a 3 fold increase from the initial zero time point. Following this initial spike in phosphorylation, which is likely due to the initial re-stimulation with serum, a subsequent increase in p-Akt (S473) began at 12 hours post release. This progressed to a maximum of an 8.6 fold increase by 24 hours post release that remained stable to 30 hours post release. Although cells exposed to lunasin also exhibited an initial spike in phosphorylated Akt at S473, this increase was considerably lower than in control cells (1.8 to 14.1 fold less at 2 to 3 hours post release respectively when compared to control cells). Also, lunasin treated cells stabilized to approximately a 3-fold increase from the initial zero time point sooner than control cells (3 and 6 hours post release respectively) and failed to exhibit a significant increase in Akt phosphorylation at 18 hours as seen in control cells (Fig. 8A-B). The onset at 12 hours post release and continued increase of Akt phosphorylation in control cells correlates with the initiation and continued decline of p27^Kip1^ levels, consistent with the negative regulation of p27^Kip1^ by p-Akt (S473). In addition, the lack of increase in p-Akt (S473) in lunasin exposed cells (other than due to initial increase due to re-stimulation with serum at 2 hours) correlates with the lack of decline in p27^Kip1^. These results suggest that lunasin inhibits the negative regulation of p27^Kip1^ through inhibiting Akt phosphorylation.

## DISCUSSION

We have demonstrated for the first time that lunasin inhibits the proliferation of NSCLC cells both *in vitro* and *in vivo*. With respect to *in vitro* growth, lunasin was significantly more active under anchorage-independent growth conditions which more closely mimic the *in vivo* environment. This was particularly striking in the case of H1299 cells, which were resistant to lunasin in adherent culture but were sensitive in colony forming as well as *in vivo* xenograft assays. This increased sensitivity in anchorage-independent assays and *in vivo* may be due to variations in the expression of cell surface receptors due to alterations in the microenvironment under these growth conditions. Given that lunasin contains a RGD domain, it is possible that changes in integrin expression are involved in the increased lunasin sensitivity. It is known that integrin expression can change dramatically in different microenvironments and that integrins are involved in the pathophysiology of NSCLC [5]. Recent studies provide evidence for the interaction of lunasin with some integrins [29, 31]. Further investigations are needed to illuminate the specificity and extent of lunasin-integrin interactions in NSCLC cells.

Mechanistic studies demonstrated that the anti-proliferative effect of lunasin on the NSCLC cell line H661 *in vitro* is not due to apoptosis or necrosis, but rather is caused by inhibition of cell cycle progression at the G1/S phase. This is in contrast to other studies that report that lunasin induces apoptosis in leukemia, mammary and colon cells with IC_50_ values of 14, 62, and 13 μM, respectively [5, 30-32, 42]. Lunasin was reported to cause G2 cell-cycle arrest and apoptosis in human L1210 leukemia cells [32] as well as in the colon cancer cell lines HT-29 and KM12L4 grown in adherent culture conditions [5, 31]. Hsieh *et al*. [30] reported that synthetic lunasin inhibits the *in vitro* growth the breast cancer cell line MDA-MB-231 with an IC_50_ of 181 μM and that lunasin-treated cells were blocked at S-phase. A later study demonstrated synthetic lunasin induced apoptosis in MCF-7 cells as well [42]. It is not particularly surprising that different cell lines respond differently to lunasin treatment given that a different complement of mutations drives the cancer phenotype in each cell line. It is also possible that differences in culture conditions or in the lunasin preparations used contributed to the disparate effects; we used a more highly purified lunasin preparation than was used in some of the earlier studies [5, 31, 32]. We found that lunasin's anti-proliferative effect in H661 cells is the result of the stabilization of p27^Kip1^levels, inhibition of cyclin D1/CDK expression levels, and ultimately, the delay and suppression of RB phosphorylation. This is consistent with the aforementioned studies on lunasin affects in colon cancer cells where cell cycle arrest was associated with increased levels of p21^Waf1/Cip1^ and p27^Kip1^. It is also consistent with the inhibition of RB phosphorylation by lunasin-like peptides isolated from *Solanum nigrum* [43].

It is well known that expression of cyclin D1, considered a delayed early gene in G1, is induced by mitogens, growth factors, and integrins and that several key regulatory pathways are precisely orchestrated to regulate the stimulatory/inhibitory signals controlling cell cycle progression [44]. These pathways include the mitogen-activated protein kinase (MAPK/ERK) pathway, the protein kinase B (Akt/PKB) pathway, the NF-κB pathway and integrin-linked kinase (ILK) signaling. Regulation of these pathways ultimately leads to cell survival and proliferation. Lunasin has been shown to suppress signaling through the FAK/ERK/NF-κB pathway in colon cancer cells [31] and Akt/NF-κB signaling in macrophages during activation of lipopolysaccharide-induced inflammation [29, 33]. These signaling effects were proposed to be mediated by lunasin interactions with α5β1 integrin in colon cancer cells and αvβ3 integrin in macrophages, albeit, no functional studies were reported to link these integrins to the observed signaling effects. Lunasin may also exert it effects through regulation of the tumor suppressor phosphatase and tensin homolog deleted in chromosome ten (PTEN). Lunasin-induced apoptosis in MCF-7 breast cancer cells were shown to be dependent on up-regulation and increased localization of PTEN to the nucleus and was independent of p53 [42]. We found that the ability of lunasin to inhibit NSCLC growth in colony-forming assays was also independent of p53 function, confirming that p53 is unlikely to be involved in modulating lunasin-induced effects. In addition to effects on the aforementioned signaling pathways that are potentially related to antagonism of integrin signaling, it is likely that lunasin's mechanism of action is multifold and may involve alterations in histone acetylation [20, 30, 43]. It is possible that there is a direct linkage between suppression of integrin signaling and histone acetylation given the recent demonstration of Akt-dependent regulation of histone acetylation in gliomas and prostate cancer [45].

A key result obtained in these studies is that lunasin inhibits the growth of NSCLC tumors in a mouse xenograft model. The fact that the cell line used to initiate the xenografts, H1299, was insensitive to lunasin in *in vitro* assays using adherent cultures clearly suggest that the microenvironment has a major effect on lunasin sensitivity. In our studies, we observed a 63% inhibition of NSCLC tumor growth at a dose of 30 mg/kg daily. This comparable to results obtained in a breast cancer xenograft model where mice were treated with 4 mg/kg or 20 mg/kg of lunasin by IP for two months prior to the implantation of MDA-MB-231 cells. Lunasin treated mice exhibited both a decrease in tumor incidence and a reduction of tumor size; 23% and 34% for the 20 mg/kg and 4 mg/kg treatments, respectively [22]. A dose of 4 mg/kg lunasin given IP has been shown reduce colon cancer metastasis by 50% in spleen implantation mouse model [31]. Taken together, these studies indicate that lunasin is a potential therapeutic for the treatment of several deadly cancers that warrants further study, particularly as an adjuvant therapy combined with other forms of treatment.

## EXPERIMENTAL PROCEDURES

### Reagents and Antibodies

All cell culture media and supplements were from Life Technologies, except fetal bovine serum (FBS) from Atlanta Biologicals; insulin, hydrocortisone, and bovine serum albumin fraction V (BSA) from Sigma-Aldrich; Bronchial Epithelial Cell Basal Medium and Bronchial Epithelial Growth Medium SingleQuots™ from Lonza; collagen from Advanced BioMatrix; and fibronectin from BD Biosciences. Primary antibodies against phosphorylated RB (Ser608, Ser780 and Ser807/811), cyclin D1, phosphorylated cyclin D1 (Thr286), CDK4, CDK6, p21^Waf1/Cip1^, p27^Kip1^, GSK-3β, phosphorylated GSK-3β (Ser9), Akt, phosphorylated Akt (S473), and β-actin were from Cell Signaling Technology. Primary antibodies against p15^INK4b^, p16^INK4a^, p18^INK4c^, p19^INK4d^and RB were from Santa Cruz Biotechnology. The phytoeryhthrin (PE)-conjugated anti-BrdU antibody and 7-aminoactinomycin D (7-AAD) nucleic acid dye were from BD Biosciences. Alkaline phosphatase (AP)-conjugated AffiniPure goat anti-rabbit IgG and horseradish peroxidase (HRP)-conjugated AffiniPure (goat anti-rabbit IgG and sheep anti-mouse IgG) were from Jackson ImmunoResearch. The nucleic acid stain 4′,6-diamidino-2-phenylindole (DAPI) was from Life Technologies. Lunasin (> 99% purity) was purified from defatted soybean flour (white flake) as previously reported by Kentucky BioProcessing [18].

### Cell Culture

Human NSCLC cells (NCI-H661, NCI-H1299, NCI-H460 and A549) and normal bronchial epithelial (NBE) cells (HBE135-E6E7 and BEAS-2B) were from American Type Culture Collection and maintained as recommended by the supplier except that A549 cells were grown in RPMI 1640 supplemented with 10% FBS, 2 mMGlutaMAX™ (L-glutamine replacement), 1 mM sodium pyruvate, 100 units/mL penicillin G sodium and 100 μg/mL streptomycin sulfate. All cultures were incubated at 37Ð C in 5% CO_2_ humidified atmosphere, routinely subcultured by traditional trypsinization and monitored for mycoplasma.

### Cell Proliferation Assays

NSCLC and BEAS-2B cells were plated in 96-well plates at 6,000 cells/cm^2^/100 μL, while HBE135-E6E7 cells were plated at 15,000 cells/cm^2^/100 μL. Cells were incubated 6 hours at 37° C in 5% CO_2_ humidified atmosphere prior to lunasin treatments (1 to 100 μM for 24, 48 and 72 hours, re-dosing every 24 hours). Vehicle (50 mM Na phosphate buffer, pH 7.4) treated cells served as negative controls. Cell proliferation was assessed using the 3-(4,5-dimethylthiazol-2-yl)-5-(3-carboxymethoxyphenyl)-2-(4-sulfophenyl)-2H-tetrazolium (MTS)-based CellTiter 96^®^ Aqueous One Solution Cell Proliferation assay kit from Promega, according to the manufacturer's instructions using a 2 hour incubation period and a Synergy™ H1 hybrid multi-mode microplate reader (BioTek^®^) to determine the absorbance.

### Anchorage-independent Proliferation Assays

NSCLC cells were plated at 6,000 cells/cm^2^/3 mL in 6-well plates in antibiotic-free growth medium and incubated 24 hours at 37° C in 5% CO_2_ humidified atmosphere. Cells were pre-treated for an additional 24 hours with vehicle or 100 μM lunasin. Anchorage-independent growth was initiated in the presence of vehicle or 100 μM lunasin. For H661 cells, cultures were harvested by trypsinization, resuspended (4,000 cells/1.25 mL/well) in 0.3% (w/v) Bacto™ agar solution (antibiotic-free RPMI 1640, 10% FBS, 2 mM GlutaMAX™) and overlaid on a 0.5 mL, 0.5% (w/v) Bacto™ agar base in 24-well plates. Cultures were incubated at 37° C in 5% CO_2_ humidified atmosphere, feeding twice weekly with antibiotic-free medium in the presence of vehicle or lunasin. After 21 days, cultures were stained with 0.005% (w/v) crystal violet and colonies (> 100 μm) imaged using an EVOS^®^ xl core inverted microscope (Life Technologies) and evaluated using ImageJ software v1.45 [46]. Anchorage-independent growth of other NSCLC cells was initiated using this method with the following modifications: H1299 cells plated at 500 cells/1.25 mL/well and incubated for 14 days, H460 cells plated at 250 cells/1.25 mL/well and incubated for 7 days, and A549 cells plated at 1,000 cells/1.25 mL/well and incubated for 21 days. All cultures were feed twice weekly with antibiotic-free medium in the presence of vehicle or lunasin.

### Apoptosis Analysis

H661 cells were plated in 8-well chamber slides at 20,000 cells/cm^2^/400 μL in growth medium and incubated overnight at 37° C in 5% CO_2_ humidified atmosphere. Cells were treated with 100 μM lunasin for 24 hours or with 1 μM staurosporine for 4 to 6 hours. Determination of apoptosis was ascertained using the Annexin V-Cy3™ Apoptosis Detection Kit (Sigma-Aldrich) according to provided instructions. Briefly, after the appropriate incubation time, cells were washed with the provided binding buffer and incubated in the double labeled staining solution (1 μg/mL Annexin V-Cy3™ (AnnCy3), 500 μM 6-carboxyfluorescein diacetate (6-CFDA), 1X binding buffer) for 10 minutes at room temperature. Cells were then washed with binding buffer, stained with 1μg/mL DAPI, washed again with binding buffer and cover-slipped. Cells were analyzed by fluorescent microscopy utilizing the Axio Observer. A1 inverted fluorescent microscope and AxioVision v4.6.3.0 software (Zeiss Microscopy).

### Cell Cycle Analysis by Flow Cytometry

H661 cells were plated at 6,000 cells/cm^2^ in 3 ml per well in 6-well plates and incubated for 24 hours at 37° C in 5% CO_2_ humidified atmosphere. Cultures were synchronized by 72 hour serum deprivation, pretreated with 100 μM lunasin for 4 hours and subsequently released in medium supplemented with 10% FBS in the presence of vehicle or lunasin. At 2 to 3 hour intervals, cultures were pulsed with 10 μM 5-bromo-2′-deoxyuridine (BrdU) for 30 minutes, harvested by trypsinization, and fixed for preparation for flow cytometry. Briefly, cells were denatured (2N HCl, 0.5% (v/v) Triton™ X-100), neutralized (0.1 M sodium borate, pH8.5) and normalized by cell counts using a hemocytometer. Cells were then labeled with PE-conjugated anti-BrdU antibody, stained with the nucleic acid dye 7-AAD and stored overnight at 4° C. Samples were analyzed by flow cytometry (10,000 events per sample) using the BD FACSAria™ and BD FACSDiva Software v6.1.2, (BD Biosciences).

### Cell Cycle Analysis by Protein Immunoblotting

H661 cells, plated at 6,000 cells/cm^2^/3 mL in 6-well plates, were incubated for 24 hours at 37° C in 5% CO_2_ humidified atmosphere, synchronized, pretreated with lunasin, and released as described for cell cycle analysis. At 2 to 3 hour intervals over a 30 hour period, cells were harvested and prepared for sodium dodecyl sulfate polyacrylamide gel electrophoresis (SDS-PAGE) and immunoblot analysis using standard methods. Briefly, cells were harvested by scraping, lysed in RIPA buffer, the protein concentration determined using the Pierce™ BCA Protein Assay Kit (Thermo Scientific) with BSA as a standard, and the total protein adjusted to 2 mg/mL in reducing sample loading buffer. Samples (7.5 μg protein/well) were subjected to SDS-PAGE, transferred by electroblotting to polyvinylidene difluoride membrane and probed with the relevant antibodies. Luminescent detection utilized the chemiluminescent substrate SuperSignal® West Femto while colorimetric detection utilized 1-Step™ NBT/BCIP (nitro-blue tetrazolium/5-bromo-4-chloro-3′-indolyphosphate) both from Thermo Scientific. Immunoblots were imaged using a Kodak Image Station 4000R Pro utilizing Carestream Molecular Imaging Software v5.0.7.24 (Carestream). Densitometry was performed using the ImageJ software v1.45.

### *In Vivo* Tumor Growth

Male, 7 – 8 week old outbred homozygous nude (Foxn1^nu^/Foxn1^nu^) mice (The Jackson Laboratory) were subcutaneously injected in the right flank with 2 × 10^6^ human H1299 cells. Two study groups comprising a vehicle control and a lunasin treatment were established (n = 10/group). The lunasin-treated group was subjected to daily intraperitoneal (IP) injections of lunasin at 30 mg/kg body weight starting upon initiation of the grafts. Tumor size was measured every other day by vernier caliper starting on day 18 through day 32. Tumor volume was calculated using the equation volume = (width)^2^ x (length)/2. Studies were carried out following established guidelines by the University of Louisville Institutional Animal Care and Use Committee (IACUC protocol #12091).

### Statistical Analysis

All data are expressed as the mean value ± SD of at least three independent experiments. Significance differences were determined using the Student's *t*-test with a probability factor *p* <0.05 being the criterion for statistical significance. Statistical analysis was performed using SigmaPlot® v11.2 (Systat Software, Inc.).
